# Data quality and timeliness of outbreak reporting system among countries in Greater Mekong subregion: Challenges for international data sharing

**DOI:** 10.1371/journal.pntd.0006425

**Published:** 2018-04-25

**Authors:** Saranath Lawpoolsri, Jaranit Kaewkungwal, Amnat Khamsiriwatchara, Ly Sovann, Bun Sreng, Bounlay Phommasack, Viengsavanh Kitthiphong, Soe Lwin Nyein, Nyan Win Myint, Nguyen Dang Vung, Pham Hung, Mark S. Smolinski, Adam W. Crawley, Moe Ko Oo

**Affiliations:** 1 The Center for Biomedical and Public Health Informatics, Faculty of Tropical Medicine, Mahidol University, Bangkok, Thailand; 2 Department of Tropical Hygiene, Faculty of Tropical Medicine, Mahidol University, Bangkok, Thailand; 3 Department of Communicable Disease Control, Ministry of Health, Phnom Penh, Cambodia; 4 Department of Disease Control, Ministry of Health, Vientiane, Lao PDR; 5 Department of Public Health, Ministry of Health and Sports, Naypyidaw, Myanmar; 6 Institute for Preventive Medicine & Public Health, Hanoi Medical University, Hanoi, Vietnam; 7 Department of Disease Control, Ministry of Health, Hanoi, Vietnam; 8 Ending Pandemics, San Francisco, California, United States of America; 9 Mekong Basin Disease Surveillance Foundation, Nonthaburi, Thailand; Baylor College of Medicine, UNITED STATES

## Abstract

Cross-border disease transmission is a key challenge for prevention and control of outbreaks. Variation in surveillance structure and national guidelines used in different countries can affect their data quality and the timeliness of outbreak reports. This study aimed to evaluate timeliness and data quality of national outbreak reporting for four countries in the Mekong Basin Disease Surveillance network (MBDS). Data on disease outbreaks occurring from 2010 to 2015 were obtained from the national disease surveillance reports of Cambodia, Lao PDR, Myanmar, and Vietnam. Data included total cases, geographical information, and dates at different timeline milestones in the outbreak detection process. Nine diseases or syndromes with public health importance were selected for the analysis including: dengue, food poisoning & diarrhea, severe diarrhea, diphtheria, measles, H5N1 influenza, H1N1 influenza, rabies, and pertussis. Overall, 2,087 outbreaks were reported from the four countries. The number of outbreaks and number of cases per outbreak varied across countries and diseases, depending in part on the outbreak definition used in each country. Dates on index onset, report, and response were >95% complete in all countries, while laboratory confirmation dates were 10%-100% incomplete in most countries. Inconsistent and out of range date data were observed in 1%-5% of records. The overall timeliness of outbreak report, response, and public communication was within 1–15 days, depending on countries and diseases. Diarrhea and severe diarrhea outbreaks showed the most rapid time to report and response, whereas diseases such as rabies, pertussis and diphtheria required a longer time to report and respond. The hierarchical structure of the reporting system, data collection method, and country’s resources could affect the data quality and timeliness of the national outbreak reporting system. Differences in data quality and timeliness of outbreak reporting system among member countries should be considered when planning data sharing strategies within a regional network.

## Author summary

The rapid growth in cross-border trade and transportation networks has led to massive population movements across borders, which increase the risk of disease spread across countries. A regional surveillance network, where disease outbreak data of each member country are shared through the network, plays an important role in prevention and control of cross-border outbreaks. However, variation in national outbreak reporting systems and guidelines used for outbreak detection and response in different countries can pose a challenge to cross-border communication for outbreak investigation. In this study, we evaluated outbreak data reported from four countries, including Cambodia, Lao PDR, Myanmar, and Vietnam, in the Mekong Basin Disease Surveillance network (MBDS). We found that the definition of outbreak varied across countries. Vietnam reported severe diarrhea outbreaks when only 1–2 cases were detected, whereas severe diarrhea outbreak reported in Myanmar consisted of more than 20 cases per outbreak. Overall, the outbreak was reported and responded within 1–15 days after the first case was detected, depending on country and how fast each disease requires public health response. Understanding outbreak reporting data would help not only to improve disease reporting systems in each country, but also improve communication and response to cross-border outbreaks among neighboring countries.

## Introduction

The rapid growth in economy and infrastructure of countries in the Greater Mekong Subregion (GMS) has brought new challenges in public health among six member countries, including Cambodia, China (Yunnan and Guangxi provinces), Lao PDR, Myanmar, Thailand, and Vietnam. Improvements in cross-border trade and transportation networks have led to massive population movements across borders, which increase the risk of disease spread across countries [1–2]. A well-established disease surveillance system within an individual country may not be sufficient for the early detection of outbreaks that arrive from neighboring countries. A regional disease surveillance network that involve collaboration among neighboring countries can assist cross-border sharing of outbreak data and human resources and expertise to control cross-border outbreaks. Several regional disease surveillance networks have been established worldwide, such as the Pacific Public Health Surveillance Network (PPHSN) among Pacific Island countries and territories, the Mekong Basin Disease Surveillance (MBDS) Network among six countries in the Greater Mekong Subregion, and the East African Integrated Disease Surveillance Network (EAIDSNet) among African countries. These networks have played an important role in the effective control of outbreaks occurring at local and global levels [3,4].

The Mekong Basin Disease Surveillance Network (MBDS) established in 1999 in collaboration with the Ministries of Health (MoH) among the six participating countries, with support from the World Health Organization and the Rockefeller Foundation [5]. The main purpose of the MBDS network is to monitor disease outbreaks and contain or prevent significant spread of infectious diseases through all neighboring countries. Disease surveillance data are required to be shared between selected border provinces through the network. However, the surveillance system of each country may vary depending on its needs and resources, which could potentially impact the integration and interpretation of outbreak information in the overall sub-region. Understanding basic data structure and data quality of outbreak reporting systems in each country is critically important to enhance the performance of cross-border outbreak detection and control.

To enable early detection of outbreaks, data on case reports must be received and reviewed in a timely manner, and analytic tools should be established to enhance recognition of potential outbreaks [6–7]. Assessing data quality is, therefore, the necessary first step to determine the capability of an outbreak detection system, including the validity of analysis results. Evaluation of the system, particularly in the aspect of data quality and timeliness, is critical to improve outbreak detection performance across the MBDS network [6–9].

Timeliness of surveillance data can affect the ability to respond rapidly and implement control measures. Timeliness can be measured using different timeline milestones common in the outbreak detection process [6, 10]. Timeliness of reporting is generally defined as the time lapse between the date of diagnosis or illness of the first case and the date that the case is reported [11–12]. Timeliness of reporting can vary according to different reporting processes (electronic or paper reports) and hierarchical structures, defined by the national surveillance guidelines [13–14]. In addition, differences in health seeking behaviors and the accessibility to healthcare services can affect the timeliness of outbreak detection and report in different countries [15]. Outbreak occurrences reported through non-governmental communication and social media may reach the public faster than traditional government reports [10, 14, 16]. However, validity of data from these informal sources remains a major concern for outbreak detection and response.

Data quality is a crucial factor to determine the validity of an outbreak detection system. Incomplete or inaccurate data can influence the detection of an unusual trend of disease occurrence [6]. Data quality can be evaluated in terms of completeness, validity of data, representativeness, and other attributes [6, 9]. For an outbreak reporting network, data quality should be evaluated according to the agreed minimum epidemiological dataset. Variables may include those that describe disease distribution in terms of time, place, and person—for example, dates at different reporting milestones such as date of onset, date of outbreak report, date of public communication, geographic distribution of the outbreak, number of cases reported, and demographic data [11–12]. Evaluation of data quality can potentially identify errors that might occur during the data collection process [6].

In the GMS subregion where outbreak reporting systems vary across countries, disease surveillance networks plays an important role as a collaborating center for data sharing and monitoring of outbreaks across borders. However, variations in reporting systems can pose a great challenge for data integration, analysis, and interpretation of cross-border outbreak scenarios. Understanding the structure and capability of reporting systems used in different countries would be useful for strengthening the disease surveillance system and data sharing among countries within the network. This study aimed to evaluate timeliness and data quality of national outbreak reporting for four countries in the MBDS network, which could potentially reflect the performance of different outbreak detection approaches in different countries.

## Methods

### Ethics statement

This study used secondary aggregated data at provincial level from public health surveillance reports. The Ministry of Health of the four countries provided permission to use the data. The project was exempted for ethical review by the Ethics Committee of the Faculty of Tropical Medicine, Mahidol University (MUTM-EXMPT 2017–004).

### Data sources

Data on disease outbreaks occurring from 2010 to 2015 were obtained from the national disease surveillance and outbreak investigation reports of each participating country, including Cambodia, Lao PDR, Myanmar, and Vietnam. China and Thailand did not participate in the study for logistical reasons. The authorities from the Ministry of Health of all participating countries set criteria and definition for minimal epidemiological data variables to be shared and analyzed. The dataset consisted of basic epidemiological information, regarding time, place, and total cases of each outbreak occurrence. Time variables included date of symptom onset in the index case (index onset), date when the number of cases reached the minimum outbreak threshold definition (threshold), outbreak detection date (detect), date of report to Ministry of Health (report), laboratory confirmation date if available (lab sent), date of initial public health response or control measures (response), date of public communication or announcement of outbreak to public by Ministry of Health about the outbreak (MOH communication), and date of report to the World Health Organization if available (report to WHO). In addition, information on geography, including state or province that the outbreak occurred, and number of suspected, probable, confirmed, and fatal cases were also included in the dataset. Even though the consensus on the selected variables was reached, it was agreed that the outbreak definition would still follow standard practices in each country, and could be different across countries. There was no attempt to standardize such information. Each member country assigned a responsible person to extract the data according to the agreed minimal epidemiological dataset in Microsoft Excel format. This information was sent to the MBDS secretariat for data integration and further analysis.

### Data analysis

The agreed minimal epidemiological dataset of each country was cleaned and reconciled. Nine diseases or syndromes with public health importance, as agreed by the authorities of the Ministries of Health of the four participating countries, were selected for analysis, including dengue, food poisoning & diarrhea, severe diarrhea, diphtheria, measles, H5N1 influenza, H1N1 influenza, rabies, and pertussis. The total number of outbreaks reported during 2010 to 2015 in each country were described. The number of confirmed cases and suspected cases reported for each disease outbreak were also summarized by year and country, using median and interquartile range.

Completeness of date data for each variable required in the dataset was defined as a non-missing date entered for that variable. Percent completeness was calculated by number of non-missing date data records divided by number of total records in each date variable. The completeness was calculated separately for each disease and country.

Data validity was measured using two methods: an inconsistent date recorded or an out-of-range date recorded. The validity was determined only for three date variables, including report date, response date, and MOH report date, since these three variables had the most complete data in all countries. The definition of epidemiological parameters for outbreak investigation are provided in Table 1. Date records were considered inconsistent if those dates were reported earlier than date of index onset, as the index onset date is considered as an initial point of the outbreak. Durations between index onset date and each of the three selected date variables (report, response, and MOH report) were also calculated. Date records that had duration > 90 days were considered as out-of-range records. Percent of invalid records was calculated by number of invalid records divided by total non-missing records. Due to the overall small proportion of incomplete and inconsistent data, the results for data quality were presented by country and disease. The annual trend of data quality was not included in the analysis.

**Table 1:** Definition of epidemiological parameters for outbreak investigation.

Indicators	Definition
Index case	The first case to be identified at the start of an outbreak.
Index onset date	Date of the symptom onset of the index case
Threshold date	Date when the observed number of cases exceeds the habitual occurrence in a defined geographical area
Outbreak detection date	Date that the outbreak was detected by public health authorities
Report date	Date that the outbreak was reported to the outbreak reporting system
Laboratory confirmation date	Date of the laboratory report for the first case within the outbreak cluster
Response date	Date when the local public health professionals took actions to control the outbreak
Public communication date	Date of the first official release about the outbreak by the Ministry of Health
WHO report date	Date that the outbreak was reported to the WHO

**Fig 1:**
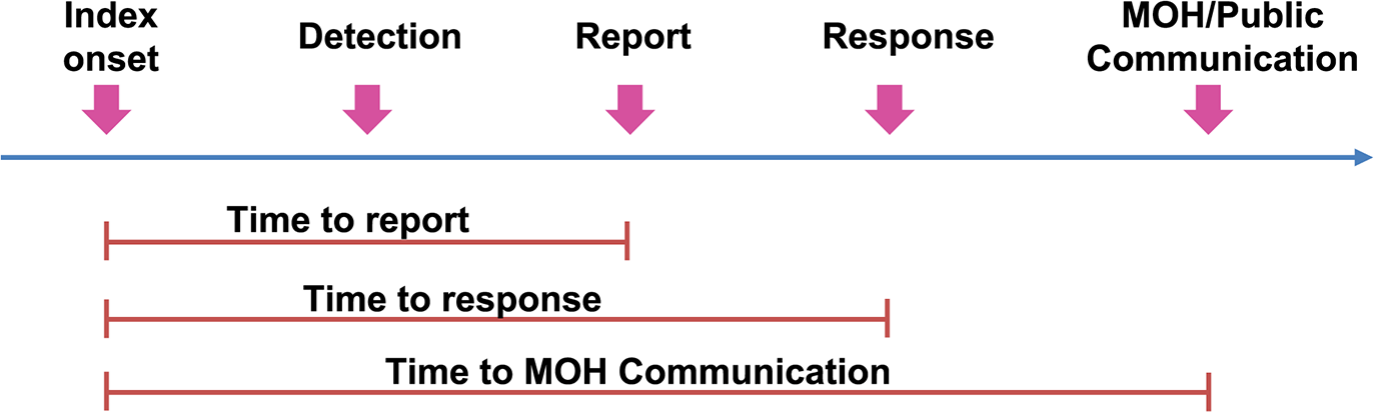
Timeline milestone for outbreak reporting system and timeliness indicators.

All valid durations were used to determine timeliness of outbreak reports. Time to report, time to response, and time to MOH report were calculated using index onset date as a reference (Fig 1). These timeliness indicators were summarized as median by year for each disease and country. Non-parametric trend analyses were performed using the Mann-Kendall test to determine the trend of timeliness indicators overtime. As the surveillance systems are different and the definitions of outbreak threshold for reporting different diseases vary across countries, it was predetermined among participating countries that comparisons between countries would not be performed. Rather, the data were analyzed and reported separately as an evidence-based assessment for each country. All analyses were performed using SAS 9.4.

## Results

### Outbreak occurrence

Overall, 2,087 outbreaks of the nine selected diseases or syndromes were officially reported in Cambodia, Lao PDR, Myanmar, and Vietnam from 2010 to 2015. The number of outbreaks varied across country and disease (Table 2). Vietnam reported the highest number of outbreaks (715 outbreaks); among those, 401 outbreaks (56%) were due to dengue infection. Myanmar reported 664 outbreaks over the 6-year period. The most common outbreaks occurring in Myanmar were food poisoning & diarrhea (212 outbreaks; 32%) and severe diarrhea (237 outbreaks; 36%). Among 288 outbreaks reported in Cambodia, 146 (51%) of them were caused by food poisoning & diarrhea. There were 420 outbreaks reported in Lao PDR; half of these were caused by diseases or conditions other than the 9 selected diseases. The common other outbreak reports included influenza-like illnesses, hand-foot-mouth disease, fever with rash, and chikungunya. Among the selected disease outbreaks, a large proportion was due to food poisoning & diarrhea (90 outbreaks; 21%) and dengue (56 outbreaks; 13%). Almost all H1N1 outbreaks were reported from Vietnam, while most rabies and pertussis outbreaks were reported from Lao PDR.

**Table 2:** Total number of outbreaks for selected diseases during 2010 to 2015 in four member countries.

	Total N (%)	Cambodia N (%)	Lao N (%)	Myanmar N (%)	Vietnam N (%)
All outbreaks	2087	288	420	664	715
Dengue	459 (22)	2 (1)	56 (13)	0 (0)	401 (56)
Food Poisoning & Diarrhea	448 (21)	146 (51)	90 (21)	212 (32)	0 (0)
Severe diarrhea	262 (13)	1 (0)	0 (0)	237 (36)	24 (3)
Diphtheria	148 (7)	1 (0)	19 (5)	38 (6)	90 (13)
Measles	125 (6)	2 (1)	0 (0)	99 (15)	24 (3)
H5N1	105 (5)	57 (20)	2 (0)	0 (0)	46 (6)
H1N1	66 (3)	0 (0)	1 (0)	0 (0)	65 (9)
Rabies	24 (1)	2 (1)	22 (5)	0 (0)	0 (0)
Pertussis	20 (1)	0 (0)	20 (5)	0 (0)	0 (0)
Other	430 (21)	77 (27)	210 (50)	78 (12)	65 (9)

The number of cases affected in each outbreak varied by disease and country (Table 3). The number of cases reported in each outbreak in Vietnam was relatively small (median of 1–2 cases) for all reported diseases, compared with other countries. A significant proportion of case reports of disease outbreaks in Cambodia and Lao PDR were suspected cases, while all cases reported in Myanmar and Vietnam were confirmed cases. For relatively rare diseases such as diphtheria and rabies, only a few cases were reported for each outbreak, whereas the median number of cases per each outbreak of food poisoning & diarrhea was ranged between 10–20 cases (Table 3).

**Table 3:** Median and interquartile of number of confirmed and suspected cases by disease outbreak and country, during 2010 to 2015.

Year	Cambodia			Lao PDR			Myanmar			Vietnam		
	# outbreaks	Confirmed cases	Suspected cases	# outbreaks	Confirmed cases	Suspected cases	# outbreaks	Confirmed cases	Suspected cases	# outbreaks	Confirmed cases	Suspected cases
**Dengue**												
2010				11	0 (0–3)	12 (8–32)				1	0	0
2011				4	3 (1–8)	24 (7–39)				2	3(2–5)	0
2012				15	2 (1–3)	23 (16–48)				91	2 (2–3)	0
2013				26	3 (1–5)	30 (18–41)				113	2 (2–2)	0
2014										37	2 (2–2)	0
2015	2	95 (37–153)	92 (37–147)							157	2 (2–3)	0
**Diarrhea and Food Poisoning**												
2010	72	18 (12–36)	15 (8–31)	8	0 (0–2)	26 (6–102)	16	8 (5–24)	0			
2011	10	20 (7–65)	18 (7–60)	12	0 (0–2)	31 (10–58)	22	19 (8–47)	0			
2012	18	14 (8–45)	13 (8–45)	28	1 (0–3)	20 (10–45)	26	15 (7–45)	0			
2013	8	14 (6–47)	14 (6–47)	20	0 (0–3)	27 (9–46)	51	15 (6–29)	0			
2014	12	10 (5–73)	10 (5–73)	12	0(0–3)	23 (14–33)	36	21 (8–50)	0			
2015	26	22 (12–37)	22 (12–36)	10	1 (0–2)	23 (8–104)	60	20 (9–62)	0			
**Severe diarrhea**												
2010							43	14 (5–30)	0			
2011							44	10 (5–15)	0	1	1	0
2012							30	21 (14–37)	0	21	1 (1–1)	0
2013							20	11 (6–23)	0	2	1 (1–1)	0
2014							57	12 (6–33)	0			
2015	1	1	0				42	16 (11–31)	0			
**Measles**												
2010							4	7 (7–10)	0			
2011							43	10 (6–23)	0			
2012							46	12 (5–20)	0			
2013							6	22 (16–48)		5	10 (9–20)	0
2014	2	1 (1–1)	0							17	5 (2–9)	0
2015										2	1 (1–2)	0
**Diphtheria**												
2010							4	1 (1–1)	0			
2011				1	0	1	6	1 (1–1)	0	3	1 (1–2)	0
2012				8	0 (0–1)	1 (1–3)	17	1 (1–1)	0	65	1 (1–2)	0
2013				5	0 (0–1)	4 (1–10)	11	1 (1–1)	0	13	1 (1–1)	0
2014										6	1 (1–2)	0
2015	1	1	0	4	5 (1–6)	23 (5–59)				3	2 (2–5)	0
**H5N1**												
2010	1	1	1									
2011	8	11 (9–14)	10 (8–13)									
2012	11	1 (1–7)	1 (1–6)	1	0	1				31	2 (1–3)	0
2013	27	10 (8–13)	9 (7–12)							11	2 (1–3)	0
2014	9	13 (9–14)	11 (8–13)	1	0	16				4	1 (1–2)	0
2015	1	2	1									
**H1N1**												
2010												
2011												
2012										42	3 (2–6)	0
2013				1	4	290				18	6 (3–35)	0
2014										4	12 (2–29)	0
2015										1	24	0
**Rabies**												
2010												
2011				3	0	1 (1–1)						
2012				4	0	1 (1–1)						
2013				8	0	1 (1–1)						
2014				6	0	1 (1–1)						
2015	2	1.5 (1–2)	0	1	0	3						
**Pertussis**												
2010												
2011				2	2.5 (2–3)	46 (29–63)						
2012				10	2 (1–9)	48 (10–84)						
2013				4	6 (6–6)	29 (16–43)						
2014				1	0	17						
2015				3	0	48 (11–124)						

### Data quality: Completeness and validity

**Fig 2:**
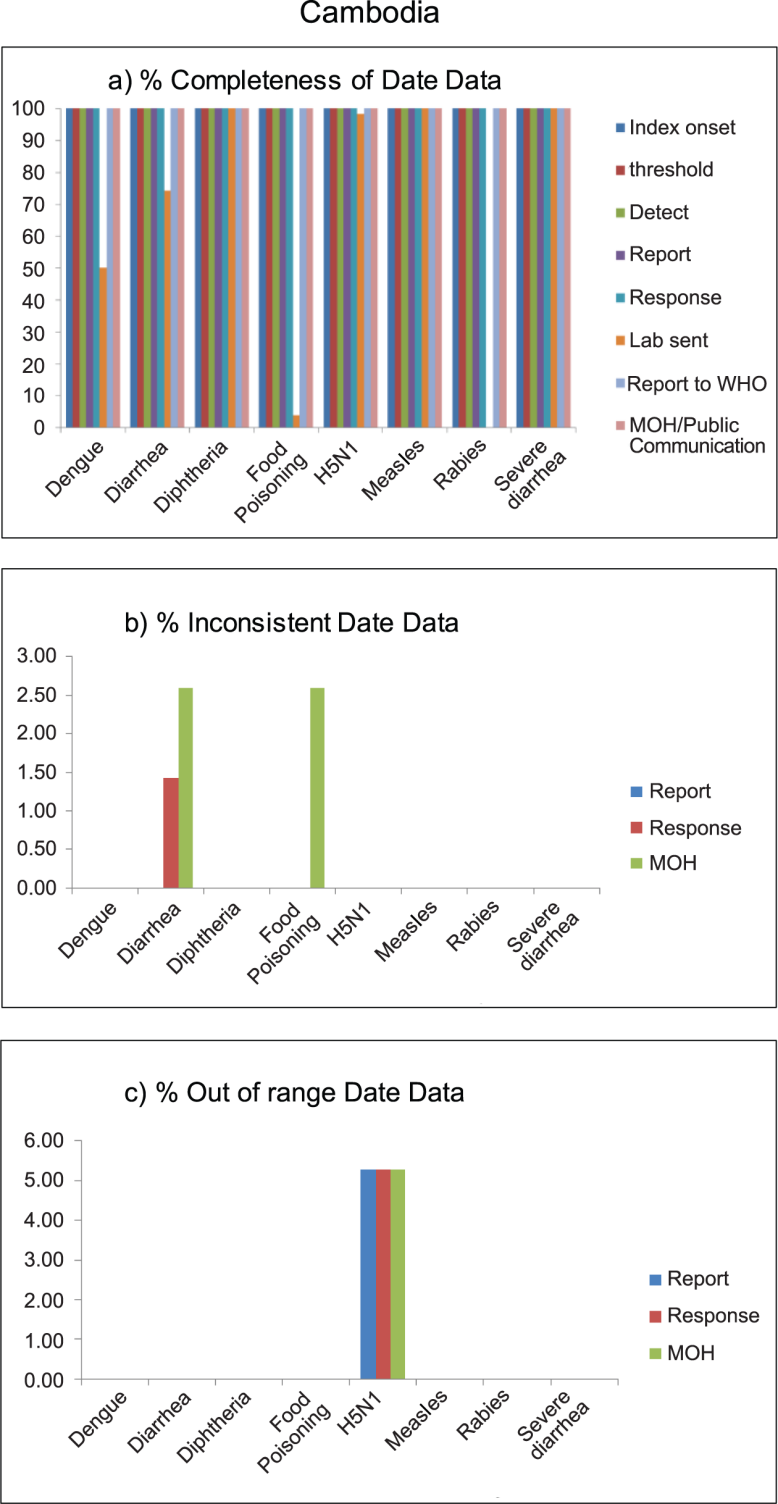
Data quality of outbreak reporting data, in terms of completeness (a), inconsistency (b), and out of range (c), by disease outbreak of Cambodia, 2010–2015.

Almost all date data in outbreak reports from Cambodia were complete. Missing data were found only in the date of laboratory confirmation, since laboratory confirmation was not sent for all diseases. Only 1% of data on response date for diarrhea outbreak was inconsistent, and 2.5% of diarrhea and food poisoning records had inconsistent in date of MOH communication. In addition, out-of-range date data was found only in H5N1 outbreak data; 5% had out-of-range report, response, and MOH communication dates (Fig 2).

**Fig 3:**
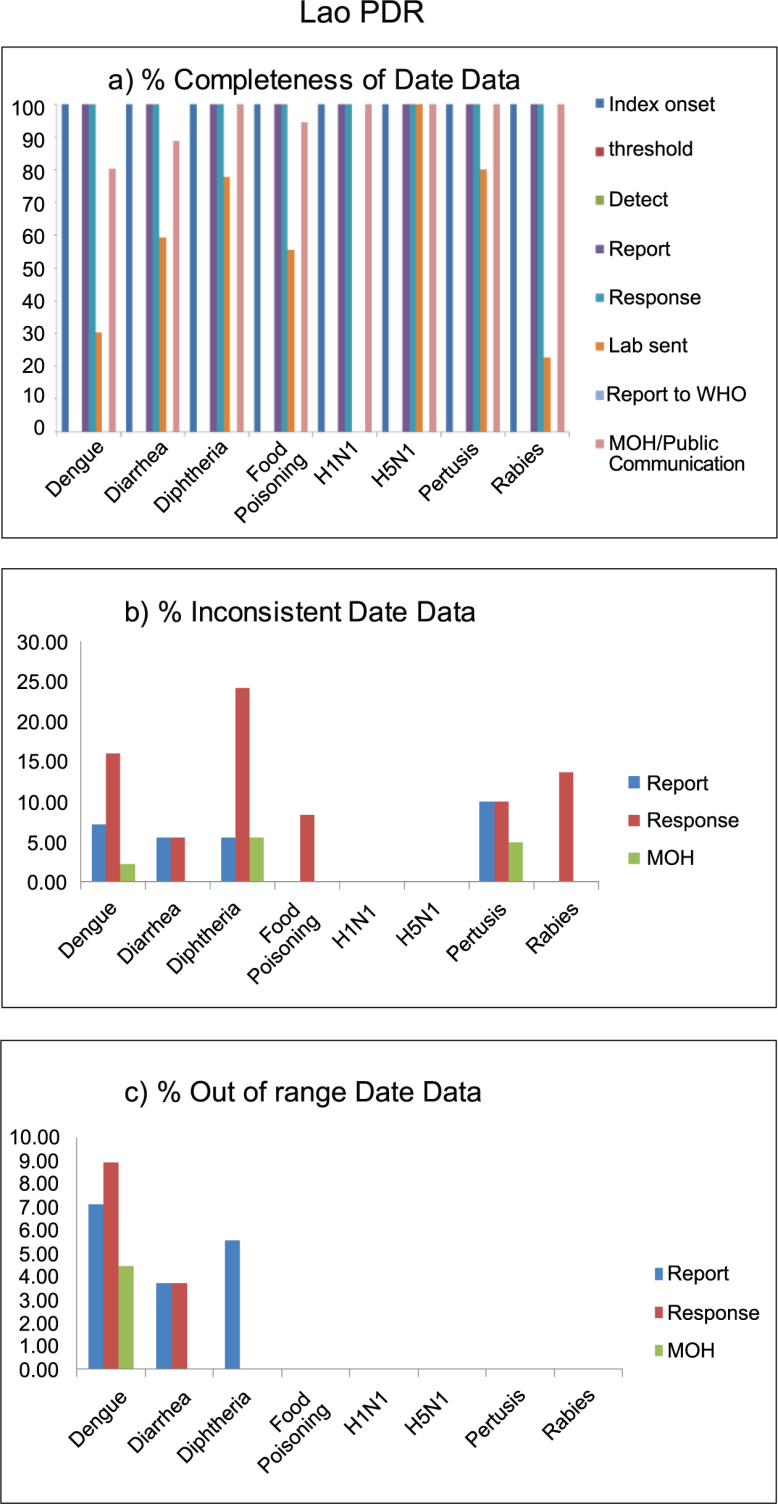
Data quality of outbreak reporting data, in terms of completeness (a), inconsistency (b), and out of range (c), by disease outbreak of Lao PDR, 2010–2015.

All outbreak reports in Lao PDR had no information on date that the outbreak reached threshold, outbreak detection date, and date when the outbreaks were reported to WHO. Similar to other countries, laboratory confirmation date was incomplete in all reported disease outbreaks, except H5N1 outbreaks for which all outbreaks had records on date of laboratory confirmation. Among those outbreak reports with non-missing date data, about 5–10% had inconsistent report, response, and MOH communication dates. Inconsistent date data were observed mostly (25%) in response date of diphtheria outbreaks. Out-of-range date data were observed <10% of three disease outbreaks, including dengue, diarrhea, and diphtheria (Fig 3).

**Fig 4:**
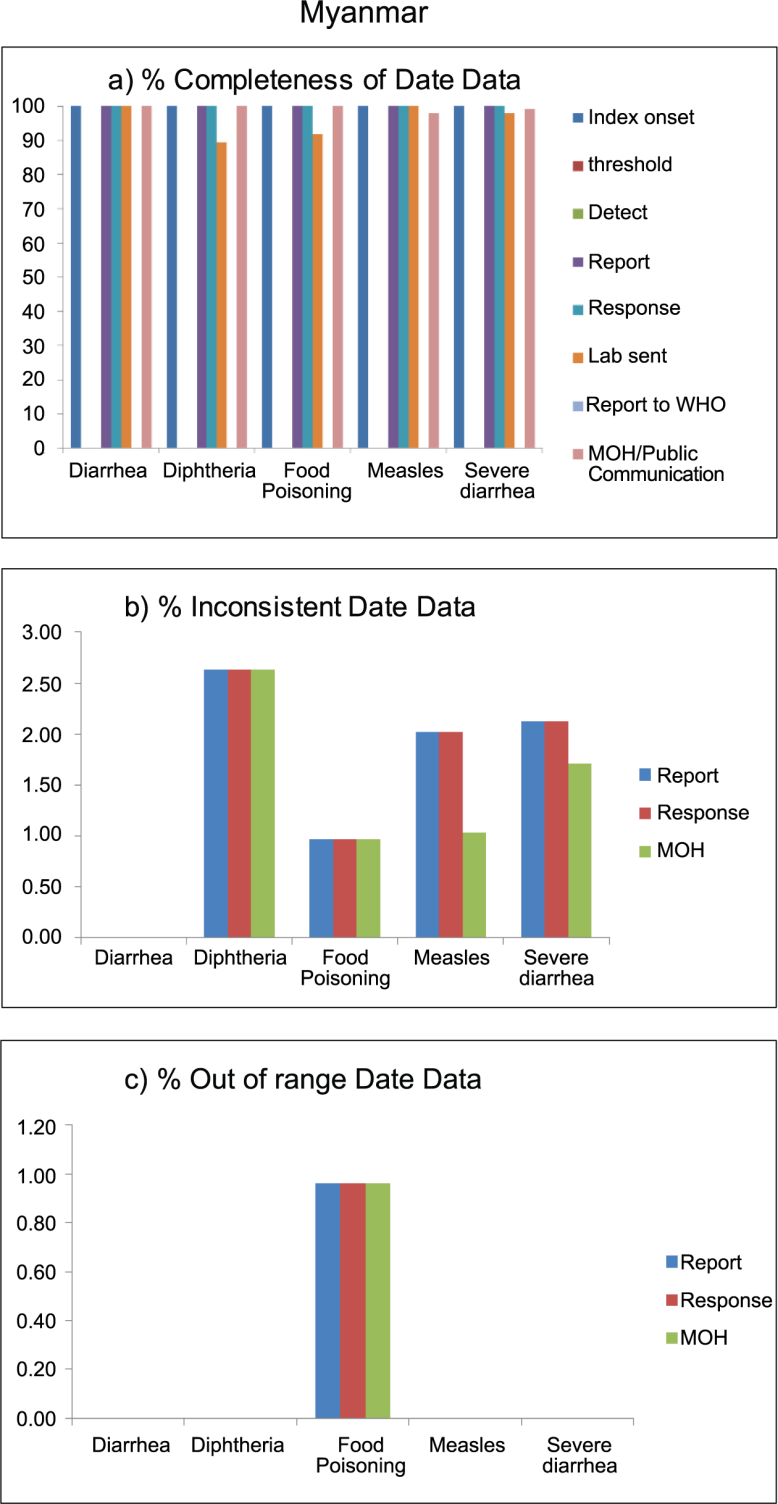
Data quality of outbreak reporting data, in terms of completeness (a), inconsistency (b), and out of range (c), by disease outbreak of Myanmar, 2010–2015.

Outbreak reports in Myanmar had no information on threshold date, detection date, and date reported to WHO. Other date variables were almost 100% complete, except diarrhea-related outbreaks, where about 90% of records had laboratory confirmation date. Approximately 1–2.5% of available date records were inconsistent; those errors were observed similarly in all three selected date variables (report, response, and MOH communication). Out-of-range date data were observed in only 1% of reports on food poisoning outbreaks (Fig 4).

**Fig 5:**
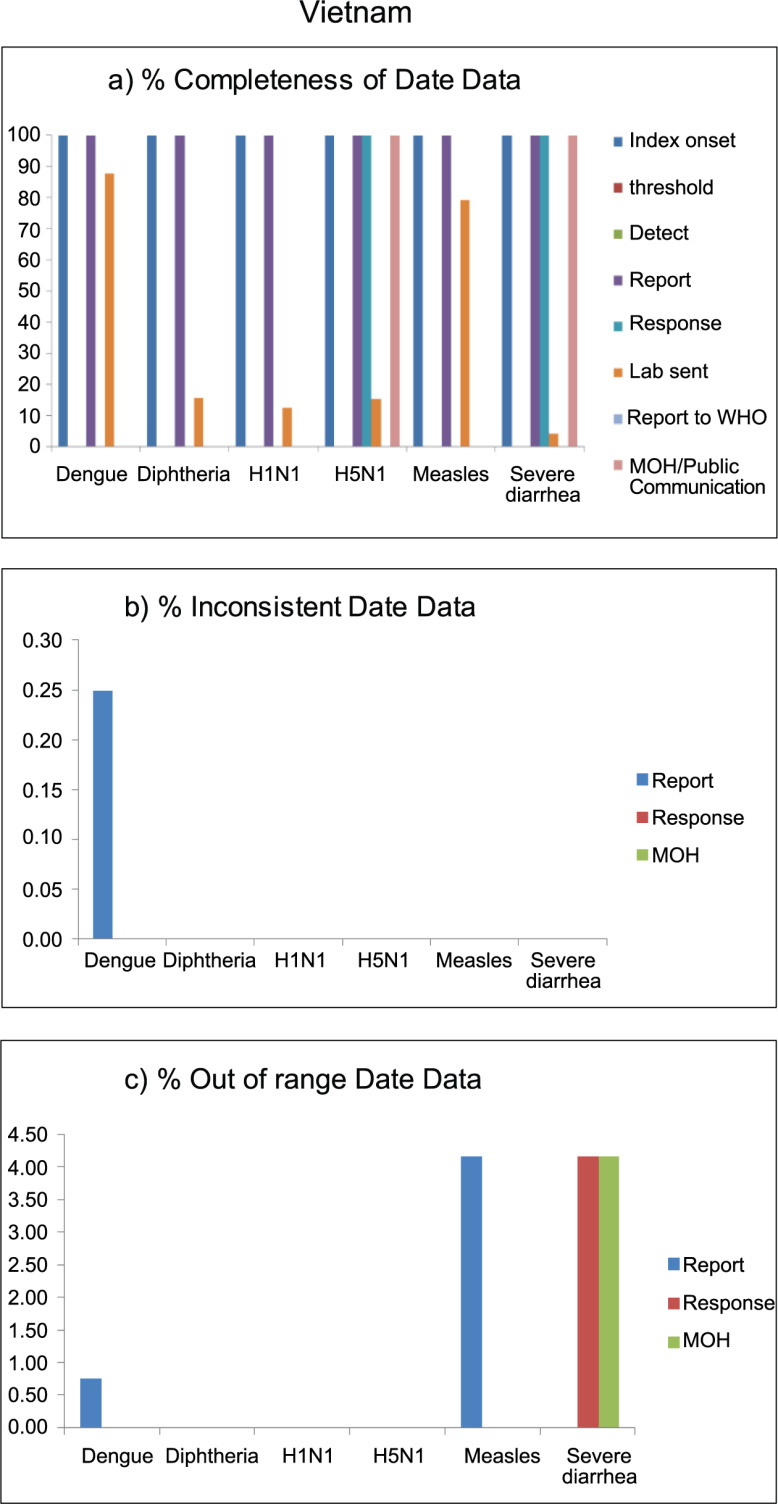
Data quality of outbreak reporting data, in terms of completeness (a), inconsistency (b), and out of range (c), by disease outbreak of Vietnam, 2010–2015.

Vietnam had few date variables included in the outbreak reports. Only index onset date and report date were 100% complete. About 80%-90% of dengue and measles outbreaks had a laboratory confirmation date, while only 10% of diphtheria, H5N1, and H1N1 outbreaks had laboratory date records. Considering the large number of dengue outbreaks reported in this country, only 0.25% (1 of 401 records) had inconsistent date of report. Inconsistent date data were not observed in other outbreak reports. Out-of-range report dates were observed in 1% and 4% of dengue and measles outbreaks, respectively, while 4% of records on response date and MOH communication date of severe diarrhea outbreaks were out-of-range (Fig 5).

### Timeliness of outbreak reports

Timeliness was calculated for three milestones (time to report, time to response, and time to MOH communication), using index onset date as a reference (Tables 4–7). Box-plots of the three timeliness indicators are provided in supplemental materials. The overall average time for report, response, and MOH communication in Cambodia ranged from 0 to 11 days (Table 4). The country responded to dengue outbreaks in <10 days, although only 2 dengue outbreaks were reported in Cambodia during the period. Regarding diarrhea & food poisoning outbreaks, the average time for report, response, and communication was about 1 day for this disease outbreak. The timeliness of report and response for diarrhea improved significantly over the period 2010 to 2015 (p-value 0.02 and 0.01). Cambodia reported only 1 severe diarrhea outbreak; this outbreak took only 3 days for the overall report, response, and communication process. Measles outbreaks were reported, responded, and communicated within 2 days. For diphtheria outbreaks, Cambodia had rapid reporting, response, and communication times, in total within 3 days, although only one outbreak was reported in 2015. The average time to the three milestones for H5N1 was relatively longer (2 to 11 days), compared with outbreak reporting for the other diseases. There was no significant improvement in the timeliness of outbreak reports for H5N1 disease over the 6-year period (2010–2015). Only two rabies outbreaks occurred in 2015, and the country’s reporting and response to the disease was rapid (within 1 day).

**Table 4:** Timeliness of outbreak report, response, and communication in Cambodia.

	Year	# outbreaks	Time to report		Time to response		Time to MOH communication	
			Median (IQR)	p-value*	Median (IQR)	p-value*	Median (IQR)	p-value*
**Dengue**								
	2015	2	6.5 (6–7)	NA	6.5 (6–7)	NA	6.5 (6–7)	NA
**Diarrhea**								
	2010	72	0.5 (0–1)	0.012	0.5 (0–1)	0.011	1 (0–2)	0.85
	2011	10	1 (0–3)		1 (0–1)		2 (1–2)	
	2012	18	0.5 (0–1)		0.5 (0–1)		1.5 (1–2)	
	2013	8	0 (0–0.5)		0 (0–0.5)		1 (1–1.5)	
	2014	12	0 (0–0)		0 (0–0)		1 (1–1.5)	
	2015	26	0 (0–1)		0 (0–1)		1 (0–1)	
**Severe Diarrhea**								
	2015	1	3	NA	3	NA	3	NA
**Measles**								
	2014	2	1.5 (0–3)	NA	1.5 (0–3)	NA	2.5 (1–4)	NA
**Diphtheria**								
	2015	1	2 (2–2)	NA	2	NA	2	NA
**H5N1**								
	2010	1	4 (4–4)	0.056	4	0.056	4	0.12
	2011	8	7 (6–8.5)		7 (6–8.5)		8 (6.5–9.5)	
	2012	11	2 (1–5)		2 (1–5)		2 (2–6)	
	2013	24	7.5 (4–11)		7.5 (4–11)		7.5 (4–11)	
	2014	9	11 (4–12)		11 (4–12)		11 (4–12)	
	2015	1	2		2		3	
**Rabies**								
	2015	2	0.5 (0–1)		0.5 (0–1)		0.5 (0–1)	

Note: No outbreak reported for H1N1 and Pertussis

*P-value based on non-parametric trend analysis (Mann-Kendall Test)

**Table 5:** Timeliness of outbreak report, response, and communication in Lao PDR.

	Year	# outbreaks	Time to report		Time to response		Time to MOH communication	
			Median (IQR)	p-value*	Median (IQR)	p-value*	Median (IQR)	p-value*
**Dengue**								
	2010	6	11.5 (7–55)	0.82	15.5 (10–57)	0.75		0.20
	2011	3	1 (0–3)		5		11 (1–12)	
	2012	6	8 (5–29)		8 (7–25)		10 (8–37)	
	2013	26	7.5 (5–11)		9 (6–12)		11.5 (9–14)	
**Diarrhea**								
	2010	4	2.5 (0.5–4)	0.15	8 (5–22.5)	0.006		0.65
	2011	12	2 (1–3)		4 (3–4.5)		11 (8.5–14)	
	2012	27	2 (1–9)		4 (2–9)		6 (3–11)	
	2013	20	3 (1.5–7.5)		4.5 (2.5–10)		7.5 (5–12.5)	
	2014	12	2 (1–3.5)		7.5 (5–10.5)		6.5 (4–9.5)	
	2015	10	6 (3–6)		9 (7–15)		9 (6–14)	
**Diphtheria**								
	2011	1	2	0.71	4	0.009	9	0.77
	2012	6	7.5 (3–9)		5 (2–40)^†^		8 (5–14)	
	2013	5	4 (4–4)		6 (6–13)		8 (7–8)	
	2015	4	5.5 (3.5–11.5)		9.5 (7–15)		8.5 (6–14)	
**H1N1**								
	2013	1	6		18		22	
**H5N1**								
	2012	1	1		2		3	
	2014	1	7		14		11	
**Rabies**								
	2011	3	3 (0–35)	0.26	7 (3–40)	0.021	7 (3–40)	0.18
	2012	4	3.5 (0.5–7.5)		2^‡^		6.5 (4–9)	
	2013	8	4 (3–6.5)		5.5 (4.5–8.5)		7.5 (6–10.5)	
	2014	6	15 (4–18)		19.5 (8–25)		18.5 (8–22)	
	2015	1	3		7		7	
**Pertussis**								
	2011	2	2 (2–2)	0.23	5 (5–5)	0.24	10 (10–10)	0.27
	2012	9	28 (12–32)		23 (14–43)		30 (15–44)	
	2013	3	29 (7–31)		33 (9–35)		35 (12–38)	
	2014	1	31		38		37	
	2015	3	25 (23–40)		29 (27–45)		28 (27–44)	

Note: No outbreak reported for severe diarrhea and Measles

*P-value based on non-parametric trend analysis (Mann-Kendall Test)

^†^ Only 2 outbreaks had valid response date

^‡^ Only 1 outbreak had valid response date

**Table 6:** Timeliness of outbreak report, response, and communication in Myanmar.

	Year	# outbreaks	Time to report		Time to response		Time to MOH communication	
			Median (IQR)	p-value*	Median (IQR)	p-value*	Median (IQR)	p-value*
**Diarrhea**								
	2009	1	1	0.056	1	0.77	1	<0.001
	2010	16	1 (1–3)		1 (1–2.5)		1 (1–3.5)	
	2011	22	2 (0–4)		2 (0–5)		4.5 (3–7)	
	2012	25	2 (1–4)		3 (2–5)		6 (4–8)	
	2013	50	1 (1–3)		2 (1–3)		4 (3–5)	
	2014	35	2 (1–3)		2 (2–4)		7 (5–9)	
	2015	59	1 (1–3)		2 (1–3)		6 (5–8)	
**Severe Diarrhea**								
	2010	43	4 (2–6)	0.011	5 (3–6)	0.007	11 (9–14)	0.002
	2011	44	5 (3–9)		6 (3–9)		11.5 (8–16)	
	2012	30	5 (3–8)		6 (4–11)		13 (10–17)	
	2013	18	4 (1–5)		5 (3–6)		10.5 (9–12)	
	2014	56	4 (1–5)		4 (1–5)		8.5 (6–11.5)	
	2015	40	2 (1–5)		4 (2–6)		10 (8–14)	
**Measles**								
	2010	4	9.5 (5–15)	0.69	9.5 (6–16.5)	0.67	21 (13.5–26.5)	0.55
	2011	43	7 (3–10		7 (5–11)		16 (11–21)	
	2012	44	6.5 (4–11.5)		7.5 (5–13)		14 (9–21)	
	2013	6	18 (6–30)		19.5 (7–32)		25.5 (12–50)	
**Diphtheria**								
	2010	4	5 (1–11.5)	0.52	7.5 (3–14)	0.51	12.5 (8–19)	0.44
	2011	6	9 (4–17)		9.5 (4–17)		16 (11–23)	
	2012	17	6 (3–7)		7 (5–10)		12 (9–15)	
	2013	10	6 (3–8)		7.5 (5–10)		12 (10–17)	

Note: No outbreak reported for dengue, H1N1, H5N1, rabies, and pertussis

*P-value based on non-parametric trend analysis (Mann-Kendall Test)

**Table 7:** Timeliness of outbreak report, response, and communication in Vietnam.

	Year	# outbreaks	Time to report		Time to response		Time to MOH communication	
			Median (IQR)	p-value*	Median (IQR)	p-value*	Median (IQR)	p-value*
**Dengue**								
	2010	1	20	0.15				
	2012	90	10 (7–14)					
	2013	113	9 (6–13)					
	2014	36	13 (9–17.5)					
	2015	157	9 (6–13)					
**Severe Diarrhea**								
	2011	1	4	0.60	4	0.60	5	0.69
	2012	21	4 (2–6)		4 (2–6)		4 (3–6)	
	2013	2	8 (3–13)		8 (3–13)		8.5 (3–14)	
**Measles**								
	2013	5	19 (15–22)	0.27				
	2014	16	16.5 (13–17.5)					
	2015	2	11 (9–13)					
**Diphtheria**								
	2011	3	6 (2–7)	0.82				
	2012	65	5 (4–8)					
	2013	13	6 (3–9)					
	2014	6	7 (3–7)					
	2015	3	3 (2–7)					
**H1N1**								
	2012	42	8 (5–10)	0.22				
	2013	18	6 (5–7)					
	2014	4	11.5 (7.5–24.5)					
	2015	1	4					
**H5N1**								
	2012	31	7 (5–11)	0.93	7 (5–11)	0.93	7 (6–11)	0.88
	2013	11	7 (6–10)		7 (6–10)		8 (7–10)	
	2014	4	6 (4–7)		6 (4–7)		6.5 (4.5–7)	

Note: No outbreak reported for diarrhea, rabies, and pertussis

*P-value based on non-parametric trend analysis (Mann-Kendall Test)

In Lao PDR, the average dengue outbreak reporting time ranged from 1 days in 2011 to 11.5 days in 2010, and it took about 3 to 11 days from outbreak report receipt to public communication. There was no significant trend in of timeliness regarding dengue outbreak data. For diarrhea & food poisoning outbreaks, the average time to report was about 2–3 days, with a time lag between outbreak reporting and communication of about 3–4 days. A significant trend in response time was observed for diarrhea outbreaks (p-value 0.006). However, the trend showed that the country took longer to response to diarrhea outbreak over the period. Lao PDR responded to diphtheria outbreaks within 10 days. Nevertheless, the time to respond to diphtheria increased significantly during 2011 to 2015 (p-value 0.009). Only 1 H1N1 outbreak occurred in Lao PDR in 2013, and the average time to report was 6 days; the time lag between outbreak report and communication was 16 days. In Lao PDR, avian influenza H5N1 outbreaks were reported in 2012 and 2014. Although only one H5N1 outbreak was reported in each particular year, the timeliness for the three milestones differed between the two years. In 2012 the H5N1 outbreak was communicated to the public within 3 days, while in 2014 the time to public communication was 11 days. Lao PDR had reported rabies and pertussis outbreaks throughout the 5-year period. Overall, the average time to report for a rabies outbreak was about 3–4 days, except for the year 2014, when rabies outbreak reporting could take up to 15 days. The time lag between report and communication of rabies outbreak was about 3 days. Lao PDR was the only country that reported pertussis outbreaks, although the majority of cases were suspected cases. The timeliness of pertussis outbreak reporting was longer, compared with reports of other disease outbreaks. On average, rabies outbreaks could take up to a month to report after index onset, except in 2011, when it took only 2 days to report the outbreak. A significant trend in timeliness indicators was not observed in rabies and pertussis outbreaks (Table 5).

Myanmar only reported outbreaks of 4 diseases—diarrhea & food poisoning, severe diarrhea, measles, and diphtheria. Myanmar’s report and response to diarrhea outbreak was rapid (within 1–2 days). However, public communication regarding the outbreak could take up to 7 days after onset of disease in an index case. Myanmar reported a large number of severe diarrhea outbreaks. The average time for report and response was about 4–6 days; and it took about 10 days for public communication by the MOH. A significant trend in improved timeliness of reporting, response, and communication for severe diarrhea outbreaks was also observed. Time to report of measles outbreaks in Myanmar was longer than other disease outbreaks reported in the country. The average time to the three milestones for a measles outbreak was 10–20 days. Diphtheria outbreaks were reported between 2010 and 2013. The overall timeliness of outbreak reporting, response and communication for diphtheria outbreaks was 6, 7, and 12 days, respectively. For diphtheria outbreaks, there was no significant trend of improved timeliness for the three milestones.

In Vietnam, data for time to report were only available for dengue, measles, diphtheria, and H1N1 outbreaks. The average time to dengue report was about 10 days in 2012–2015. Vietnam had reported, responded, and communicated severe diarrhea outbreaks within 4–8 days during the year 2013 to 2015. The average time to report of measles outbreaks was relatively longer (>10 days) than the time to report other diseases in this country. In Vietnam, diphtheria outbreaks were usually reported in <10 days after index onset. For H1N1 outbreaks, the average time to report was within 11 days. For avian influenza H5N1 outbreaks, the timeliness for the report, response, and public communication milestones was < 8 days. The temporal trend in the timeliness of the three milestones was not significant for all reported diseases.

## Discussion

Cross-border and inter-country outbreaks are a major public health threat worldwide [17–19]. Regional disease surveillance networks play an important role in effectively monitor and control cross-border disease outbreaks [20]. Differences in language, culture, surveillance system and political engagement among member countries are the major challenges in harmonizing surveillance data across countries [12, 20]. The Mekong Basin Disease Surveillance Network (MBDS) is one of the subregional networks that was established based on the mandated Memorandums of Understanding (MOUs) among governments of six member countries in the Greater Mekong Subregion. The network acts as a coordinator for cross-border and inter-country outbreak monitoring and investigation. Difference in surveillance system structure could potentially lead to differences in quality of outbreak data among member countries, which create difficulty in integration, analysis, and interpretation of outbreak surveillance at subregional level. In this study, data from the national outbreak reporting systems of four member countries in MBDS were explored to evaluate data quality and timeliness of the reporting system.

Among 2,087 outbreaks reported for the selected 9 diseases and conditions, half were due to dengue and diarrhea, suggesting that dengue and diarrhea are the main public health problems in this region. Cross-border outbreaks of the two diseases could potentially occur. A strong collaboration in the MBDS network could play an important role in the investigation and control of outbreaks among neighboring countries [17, 21].

In general, outbreaks are recognized when the observed number of cases exceeds the habitual occurrence in a defined geographical area. Therefore, the definition of “outbreak” may vary across countries, resulting in variation in the number of accumulated cases that reach outbreak threshold across place and time. Understanding the definitions of “case” and “outbreak” used in neighboring countries would be helpful, particularly for cross-border outbreak communication. In Vietnam, only a few cases were reported in each outbreak, while other countries reported more cases per outbreak. Countries typically develop reporting protocols to be used within the country, for example requiring laboratory confirmation for case reporting, reporting timeline milestone, and information required for the report. In Lao PDR, most of the outbreak reports contained a relatively large number of suspected cases, compared with the number of confirmed cases, whereas, most outbreak reports from Vietnam included only confirmed cases. However, the accuracy of the number of cases reported in each outbreak was not evaluated in this study. It is unusual to have an outbreak with zero cases reported, as observed in a dengue outbreak reported in Vietnam in 2010. The number of cases occurring in each outbreak may affect the performance of outbreak reporting and response, including data quality and timeliness. Due to differences in case definition and defined outbreak threshold, this study did not aim to compare the evaluation of outbreak reporting system in different countries. However, it would be difficult for data sharing and interpretation of outbreak at regional level if different case and outbreak definitions were used among member countries. Therefore, case and outbreak definition should be discussed when planning data sharing strategies within the network [12]. Moreover, enhancing laboratory capacity within a country would improve the validity of outbreak report at both country and network level.

Regarding data quality, this study found that the completeness of date data varied across countries. This could reflect different outbreak reporting structures among the four countries. Almost all countries did not record the date that the outbreak threshold was reached. Discussion with key persons in each country revealed that threshold date is usually the same as the report date. Therefore, recording threshold date may not be necessary. Incomplete data were also observed for the date that specimens were sent for laboratory confirmation. However, this could be a normal occurrence since laboratory confirmation may not be practical in all suspected cases, especially in limited resource settings. Interestingly, in all countries, the data were almost 100% complete for variables that the country routinely recorded, such as report and response dates. This evaluation of data completeness suggested that the minimal epidemiological dataset should be carefully discussed within the network, considering different standard practices and reporting structures among the member countries. However, even if the minimal standard dataset was agreed, incomplete reports could still be observed, particularly during the initiation of network surveillance [10].

Inconsistent and out-of-range date data were observed in about 1%-5% of report, response, and MOH communication date variables across different diseases/syndromes. These out-of-range records could either be data entry errors or the actual delayed reports. A relatively large percentage of inconsistent date data was observed in data of Lao PDR, where a paper-based reporting system is still used in some areas. Paper-based reporting systems may be prone to errors. Electronic data entry, as used in other countries, can allow for validation that reduces illogical date sequences among different variables [22]. However, considering that each country has one reporting system that applied to all disease outbreaks occurred within the country, the intra-country variation in data quality across different diseases could be the actual delayed reports. Although the intra-country variation observed in this study was only 1%-5%, further investigation is needed to identify the actual cause of the out-of-range date data.

The overall timeliness of report, response, and public communication for the outbreak was within 10–15 days in all countries. A previous study that evaluated time from index case onset to public communication from governmental outbreak reports during 1996–2009 found the estimated median time to outbreak communication in 11 countries in Southeast Asia was about 28 days; the time for reporting and communication tended to decreased over time during the 14-year period [10, 16]. The inconsistency in timeliness between our study and the previous study could be explained by the different study duration and the number of countries included in the analysis. In this study, the significant improvement in the timeliness of outbreak reporting was only observed in diarrhea and severe diarrhea outbreaks reported in Cambodia and Myanmar. In the two countries, diarrhea outbreaks were common; public health staff may have more experiences in outbreak investigation protocol for diarrhea and severe diarrhea. Technological advancement in the surveillance systems is a factor that could influence the timeliness of outbreak investigation [16]. In this study, the surveillance systems of the four countries did not change over the study period. Therefore, the overall temporal trend in the timeliness was not consistently observed in this study. Although outbreak investigation and response protocols of all member countries were developed following the WHO recommendation, the detailed activities may vary across countries depending on availability of human and financial resources. The variation in outbreak investigation and response activities among countries could affect the timeliness of outbreak response and communication.

However, Cambodia tended to have rapid reporting and response to an outbreak after index onset. Cambodia has integrated an event-based reporting tool into the national outbreak reporting system, which allows community participation in notifying unusual occurrences. Information outside the public health system, including community notification and social media, has been shown to improve the timeliness of outbreak detection and report [14]. In addition, the electronic reporting system used in Cambodia can reduce the complex hierarchical structure of the reporting system, which potentially improves the timeliness of reporting data [14].

Timely detection and reporting of outbreaks requires human and financial resources [11]. In limited resource settings, countries may need to prioritize diseases with public health importance that require close monitoring. Findings from this study also revealed that the timeliness of reporting systems varied across diseases. In this region, dengue and diarrhea related diseases are the main public health problems. Findings of this study indicated that diarrhea and severe diarrhea outbreaks had the most rapid time to report and response, whereas, some diseases such as rabies, pertussis, and diphtheria required a longer time to report and respond. Acceptable time to report and response may be defined differently according to disease transmission patterns. Diarrheal outbreaks would require rapid investigation and response to control the outbreak, while immediate response to rabies disease may not be that crucial to stopping the further spread of an outbreak. Further study is suggested, to explore the underlying factors for variation in the timeliness of reporting and response for different diseases.

The main challenge for this study was the data integration process. Although all four countries had shared the outbreak data according to the agreed variables, a number of data reconciliation process was required before data integration due to different date formats and disease name used among the countries. Differences in date format and units are common problems for data integration from multiple sources [23]. To facilitate interoperability among different systems, data and transmission standards should be implemented within the network [6].

In this study, only data quality and timeliness indicators were evaluated; as these two indicators are crucial for an outbreak reporting system for rapid detection and response to outbreaks. However, other evaluation indicators, such as flexibility, representativeness, security, and system stability, should be determined for a more complete evaluation of disease surveillance systems. In addition, outbreak data of only four out of six member countries were included in the study. China and Thailand did not participate in this study for logistical reasons. Although this study aimed to evaluate the outbreak reporting systems of each individual country, the missing information from the two member countries could affect the representativeness of the findings of the present study.

Timeliness of outbreak report and response does not reflect solely on the performance of the outbreak reporting system; it could also reflect the performance of healthcare services in the country, including health seeking behavior and accessibility to healthcare [15]. Since information on the capability of healthcare services in each country was not explored in the study, it would be difficult to conclude whether delayed reports were due to the outbreak reporting system or the healthcare services provided in each country. Both technical and healthcare structure should be considered to improve the outbreak surveillance system.

In conclusion, this study provided an insight into the data quality and timeliness of the outbreak reporting systems in different countries in the Greater Mekong subregion. The performance of an outbreak reporting system, particularly data quality and timeliness, can be affected by different factors, including the availability of human and financial resources, the hierarchical structure of the surveillance system, data collection method (paper-based or electronic-based), and healthcare services in the country. This information is not only useful for individual countries to strengthen their current surveillance system, but also important for strategic planning for data sharing within and between countries. Data sharing on disease outbreaks is a crucial part of any regional disease surveillance network to monitor and control cross-border disease outbreaks effectively. Integration of outbreak data, based on agreed minimal epidemiological dataset, would be helpful to strengthen the existing network. However, differences in data structure, data quality, and reporting systems among different countries should be considered when using integrated data and when sharing data between all countries. In addition, implementation of data and information transmission standards throughout the network would be helpful for the data integration process. It is quite a challenge, or perhaps even impossible, to have the same outbreak threshold and reporting mechanisms across countries within the network, but it would be ideal to have a common data sharing platform for each country to provide outbreak information regardless of common case/outbreak definitions. It would need collaborative effort and formal agreement among authorities in the country network to share such outbreak detection information of their own country in a timely manner to prevent and control the diseases within and across country borders in the region and beyond.

## Supporting information

S1 Checklist STROBE checklist. (DOCX)

S1 Fig Box-plot of duration between index onset date and report date, response date, and MOH public communication: Dengue outbreaks. (TIF)

S2 Fig Box-plot of duration between index onset date and report date, response date, and MOH public communication: Diarrhea and Food Poisoning outbreaks. (TIF)

S3 Fig Box-plot of duration between index onset date and report date, response date, and MOH public communication: Severe diarrhea outbreaks. (TIF)

S4 Fig Box-plot of duration between index onset date and report date, response date, and MOH public communication: Measles outbreaks. (TIF)

S5 Fig Box-plot of duration between index onset date and report date, response date, and MOH public communication: Diphtheria outbreaks. (TIF)

S6 Fig Box-plot of duration between index onset date and report date, response date, and MOH public communication: Influenza H1N1 outbreaks. (TIF)

S7 Fig Box-plot of duration between index onset date and report date, response date, and MOH public communication: Avian influenza H5N1 outbreaks. (TIF)

S8 Fig Box-plot of duration between index onset date and report date, response date, and MOH public communication: Rabies outbreaks. (TIF)

S9 Fig Box-plot of duration between index onset date and report date, response date, and MOH public communication: Pertussis outbreaks. (TIF)
