# Supplementary figures and images for: Data quality and timeliness of outbreak reporting system among countries in Greater Mekong subregion: Challenges for international data sharing
Source: PLoS Negl Trop Dis. 2018 Apr 25;12(4):e0006425. doi: 10.1371/journal.pntd.0006425 (PMC5937798; doi:10.1371/journal.pntd.0006425)

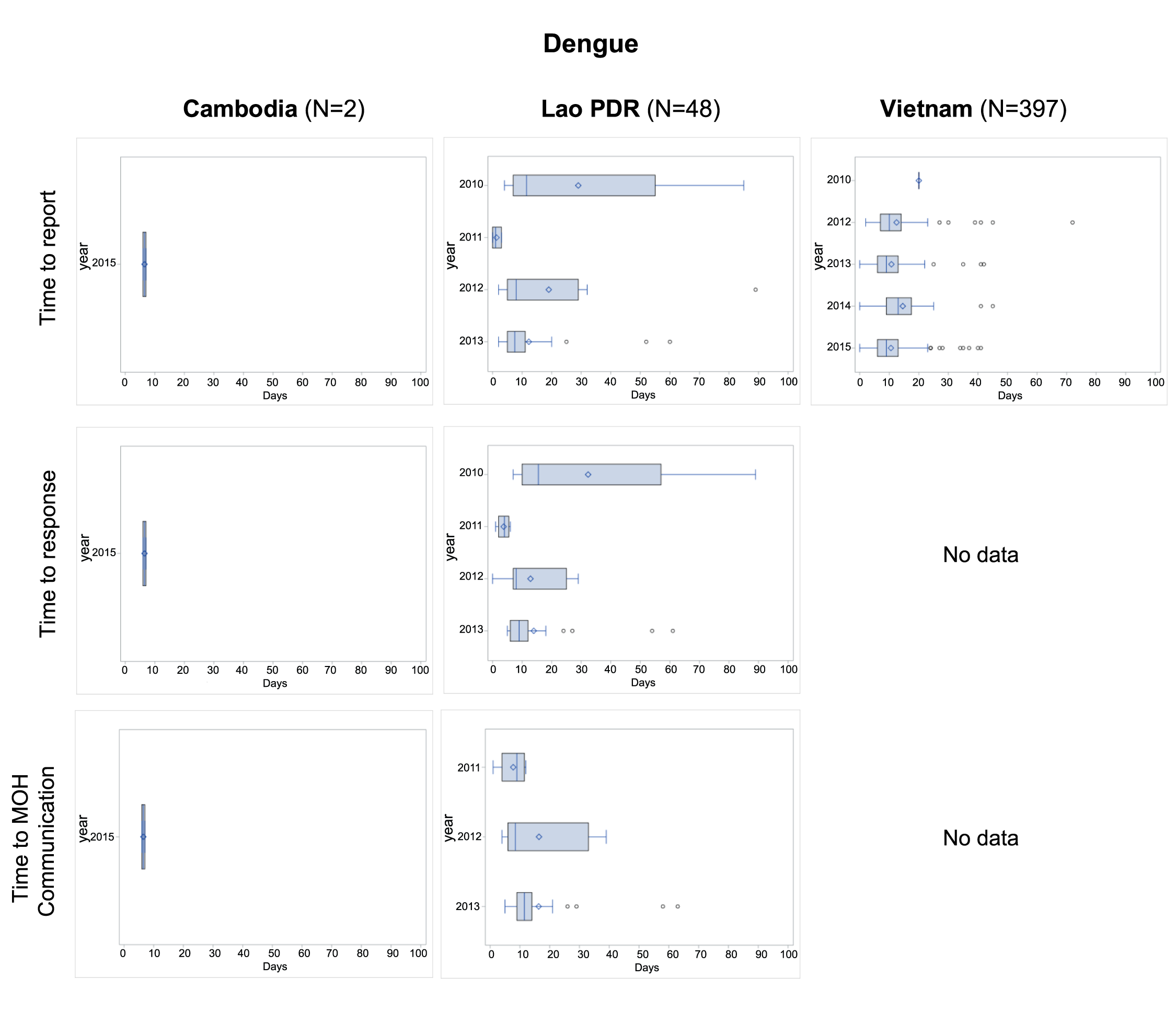

Supplement: S1 Fig — (TIF) [file pntd.0006425.s002.tif]

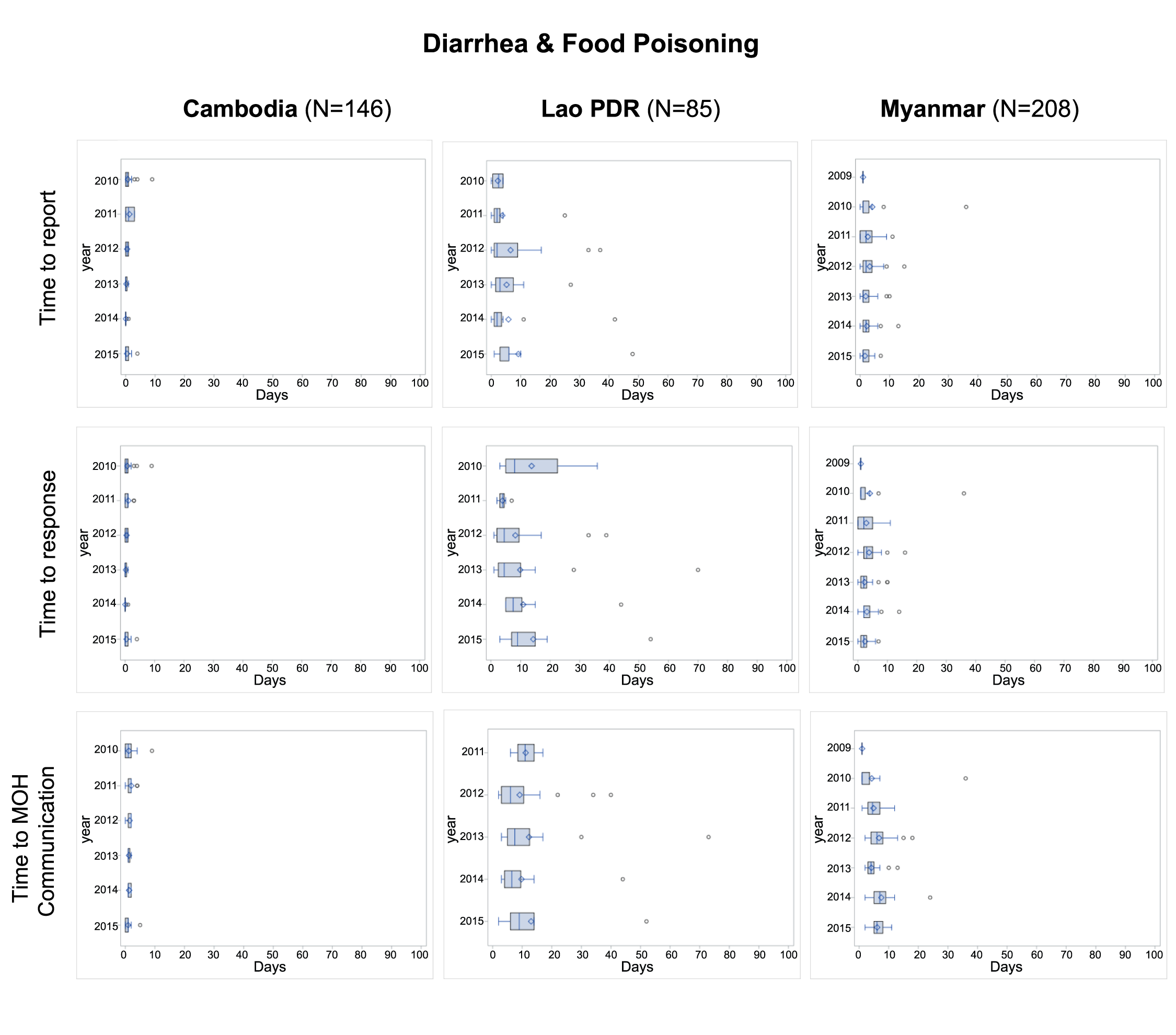

Supplement: S2 Fig — (TIF) [file pntd.0006425.s003.tif]

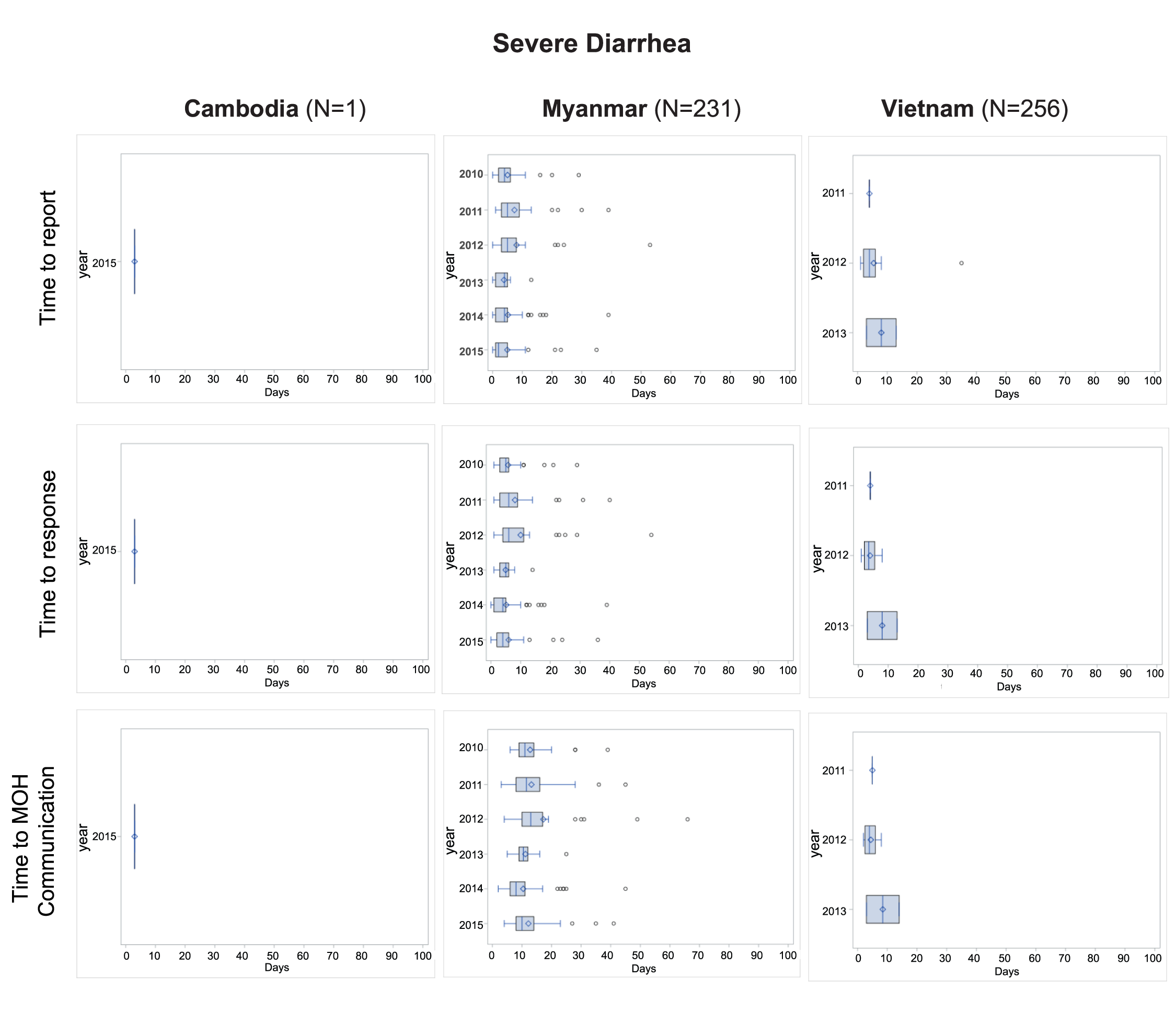

Supplement: S3 Fig — (TIF) [file pntd.0006425.s004.tif]

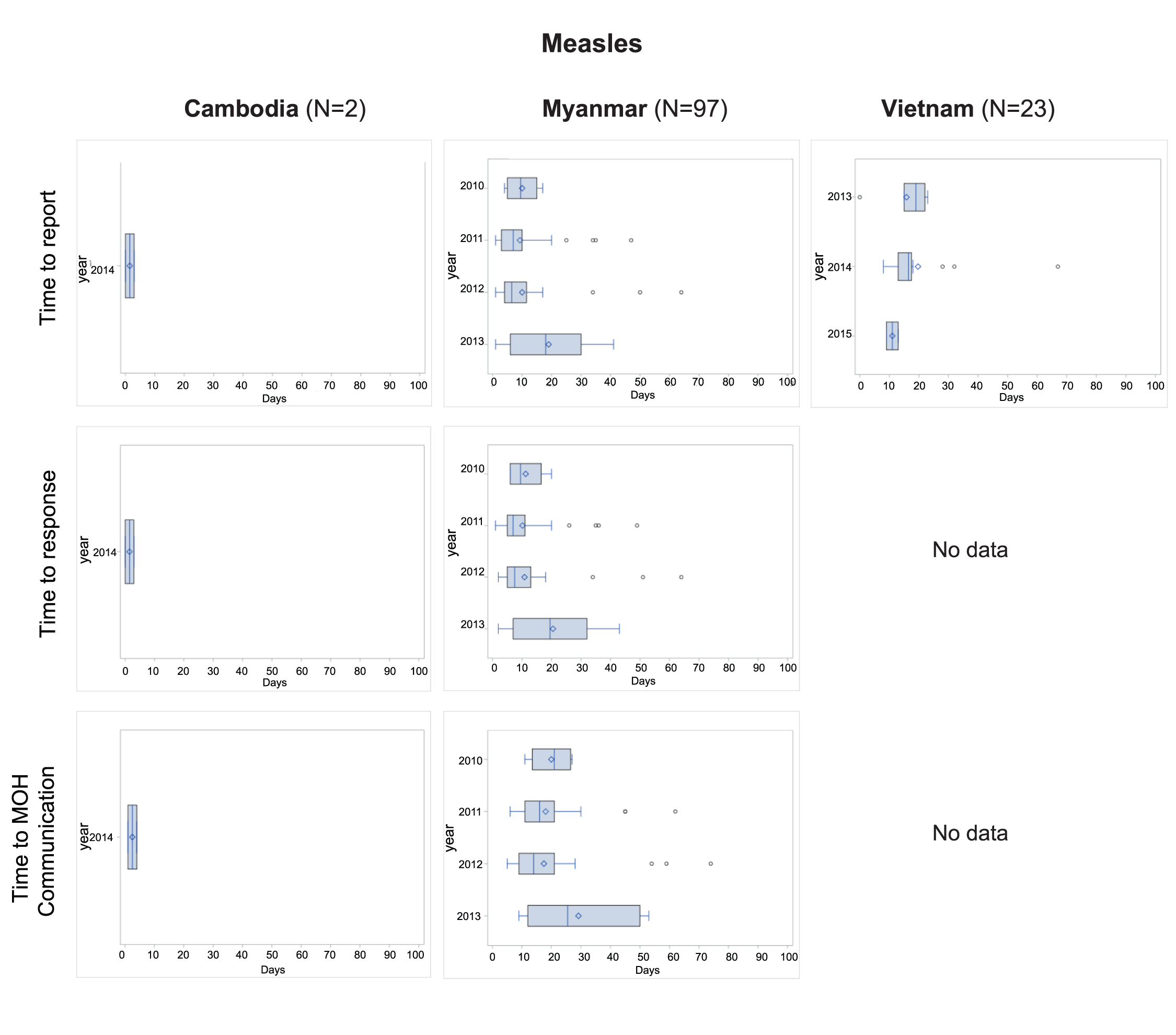

Supplement: S4 Fig — (TIF) [file pntd.0006425.s005.tif]

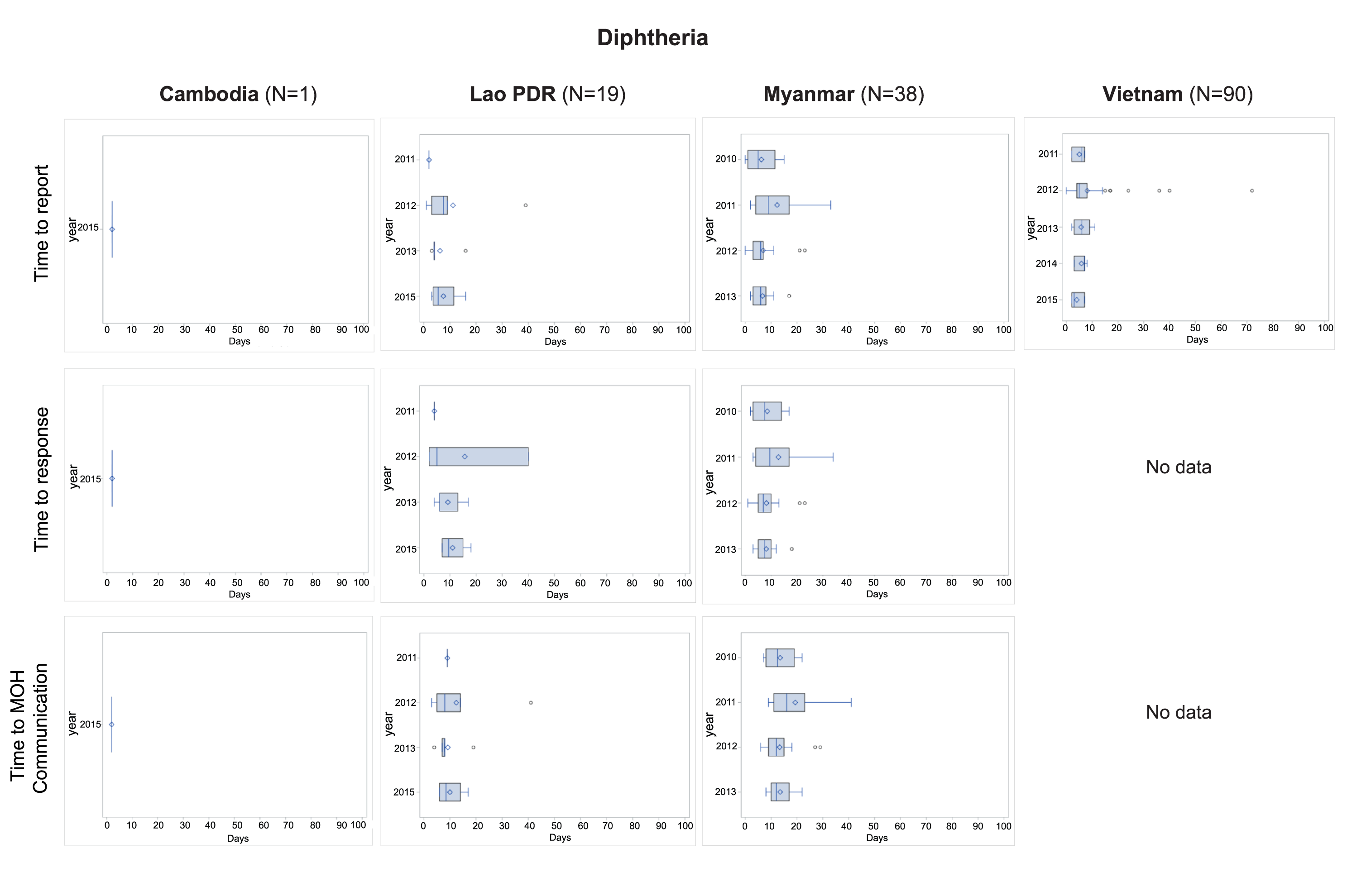

Supplement: S5 Fig — (TIF) [file pntd.0006425.s006.tif]

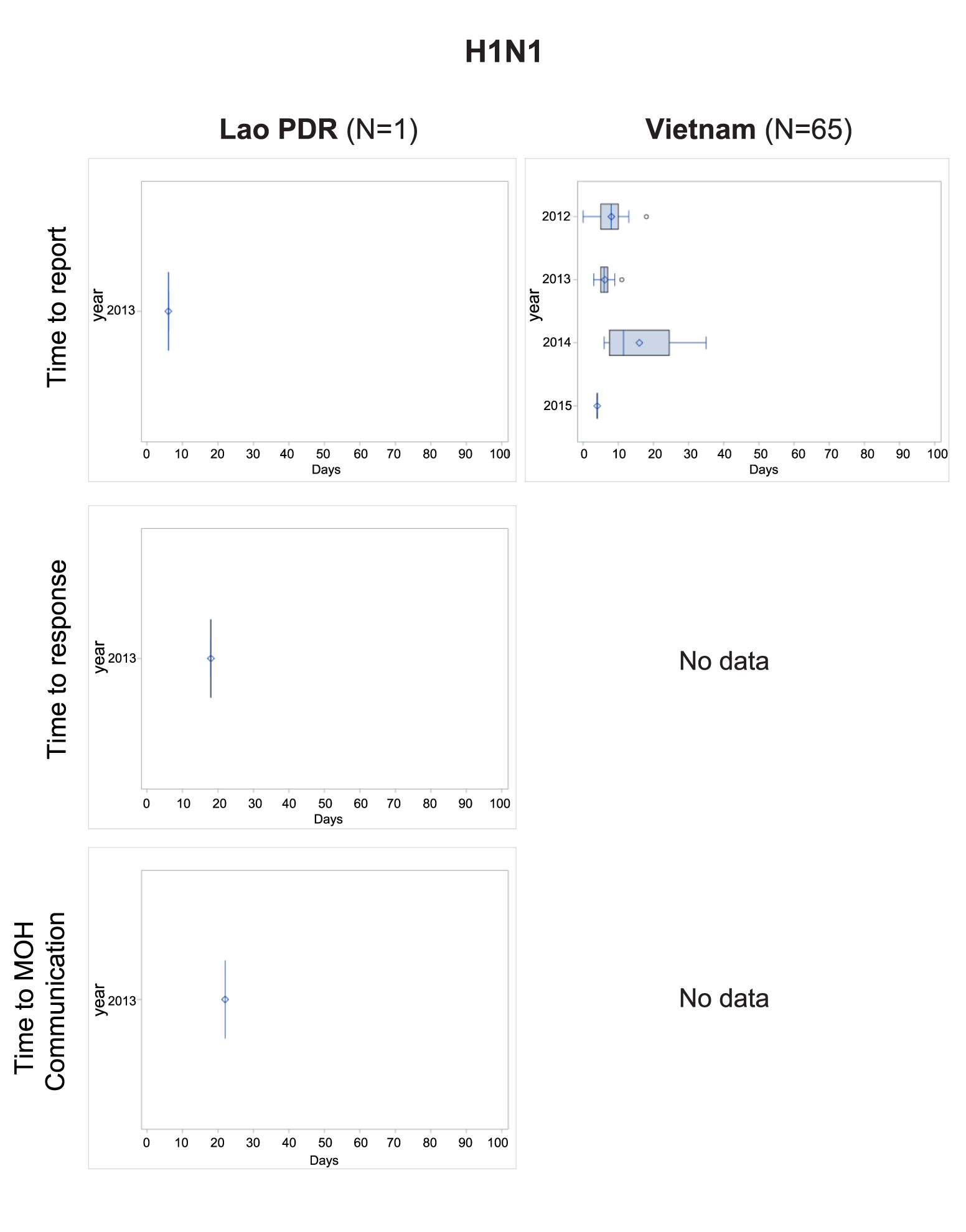

Supplement: S6 Fig — (TIF) [file pntd.0006425.s007.tif]

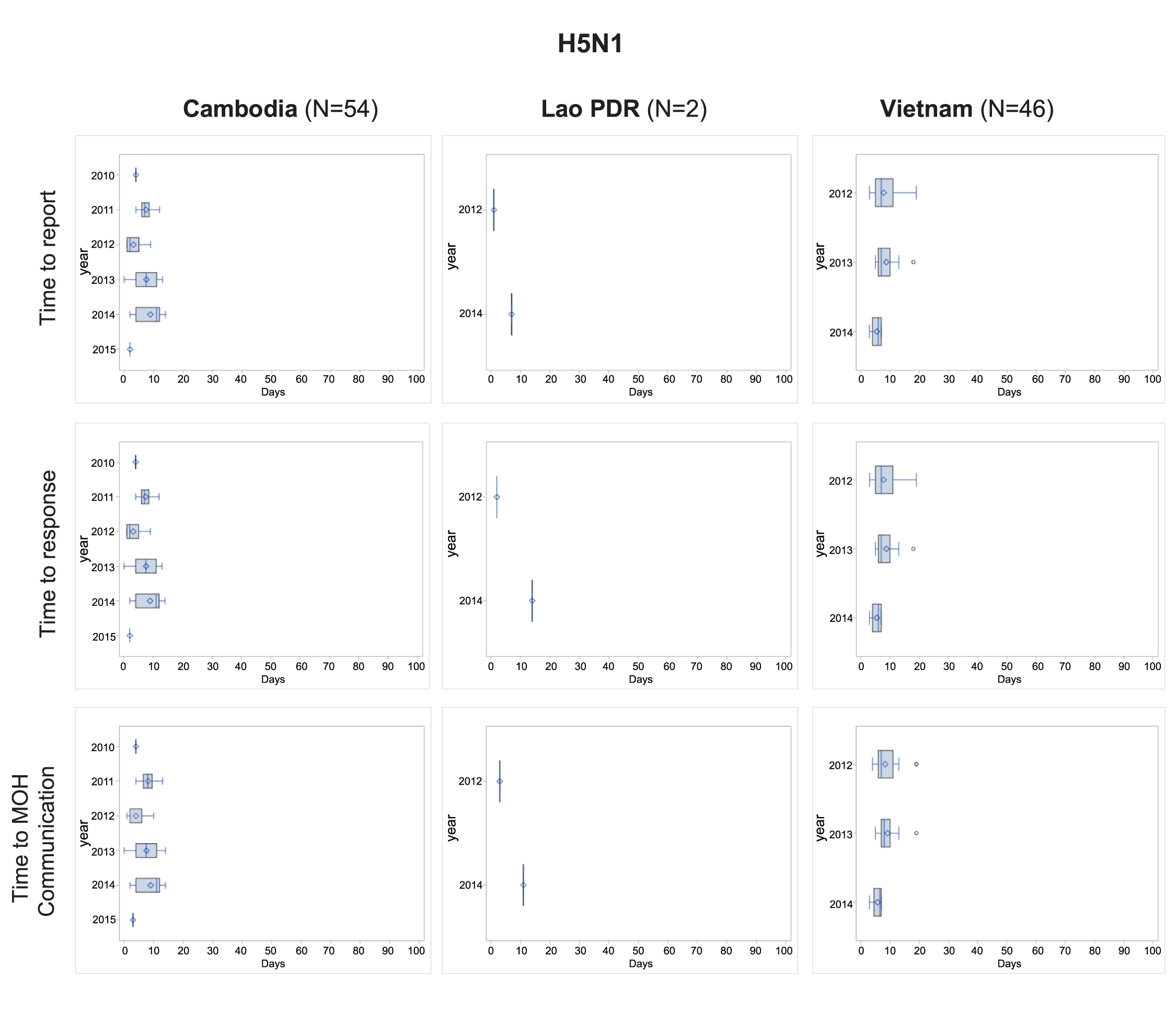

Supplement: S7 Fig — (TIF) [file pntd.0006425.s008.tif]

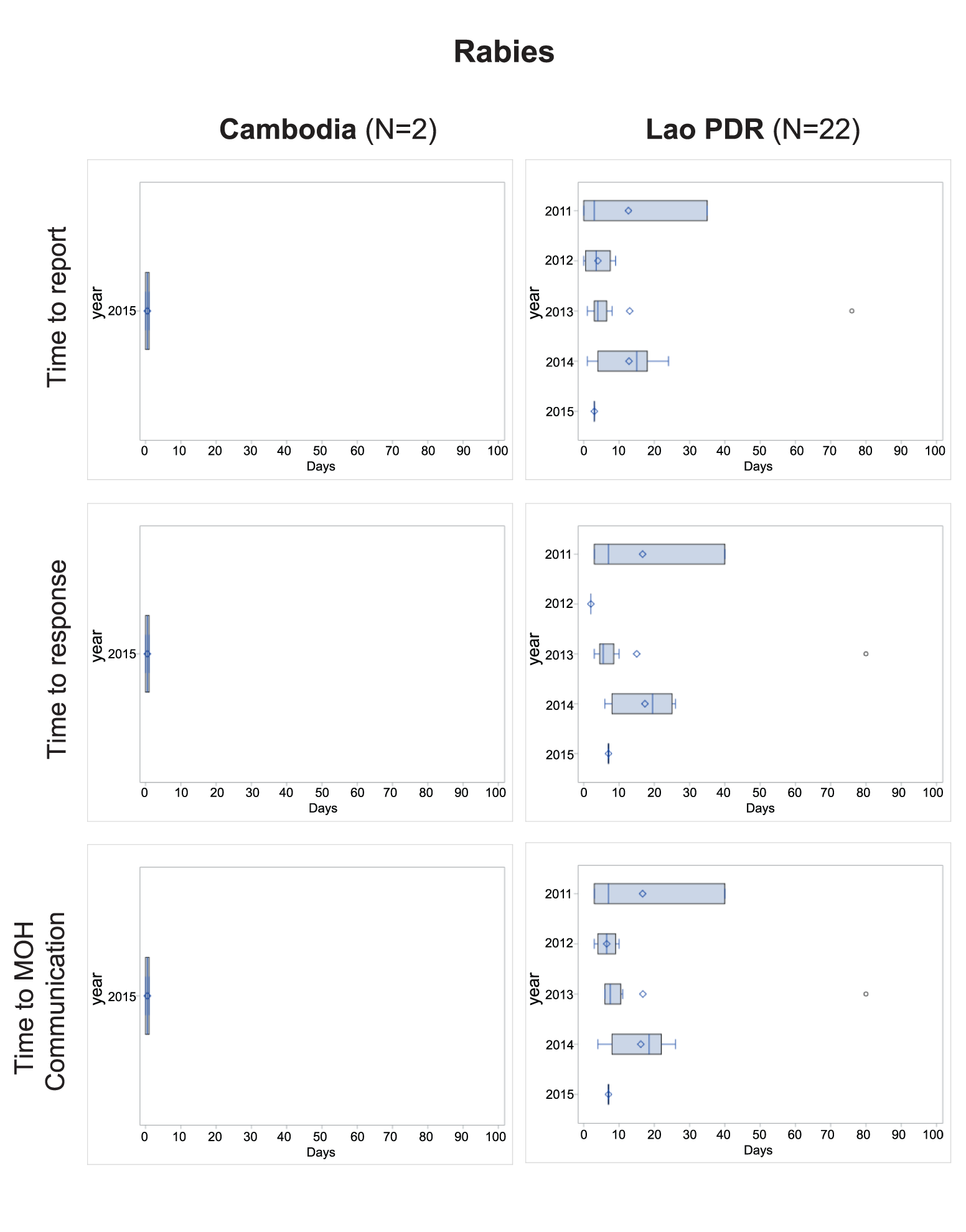

Supplement: S8 Fig — (TIF) [file pntd.0006425.s009.tif]

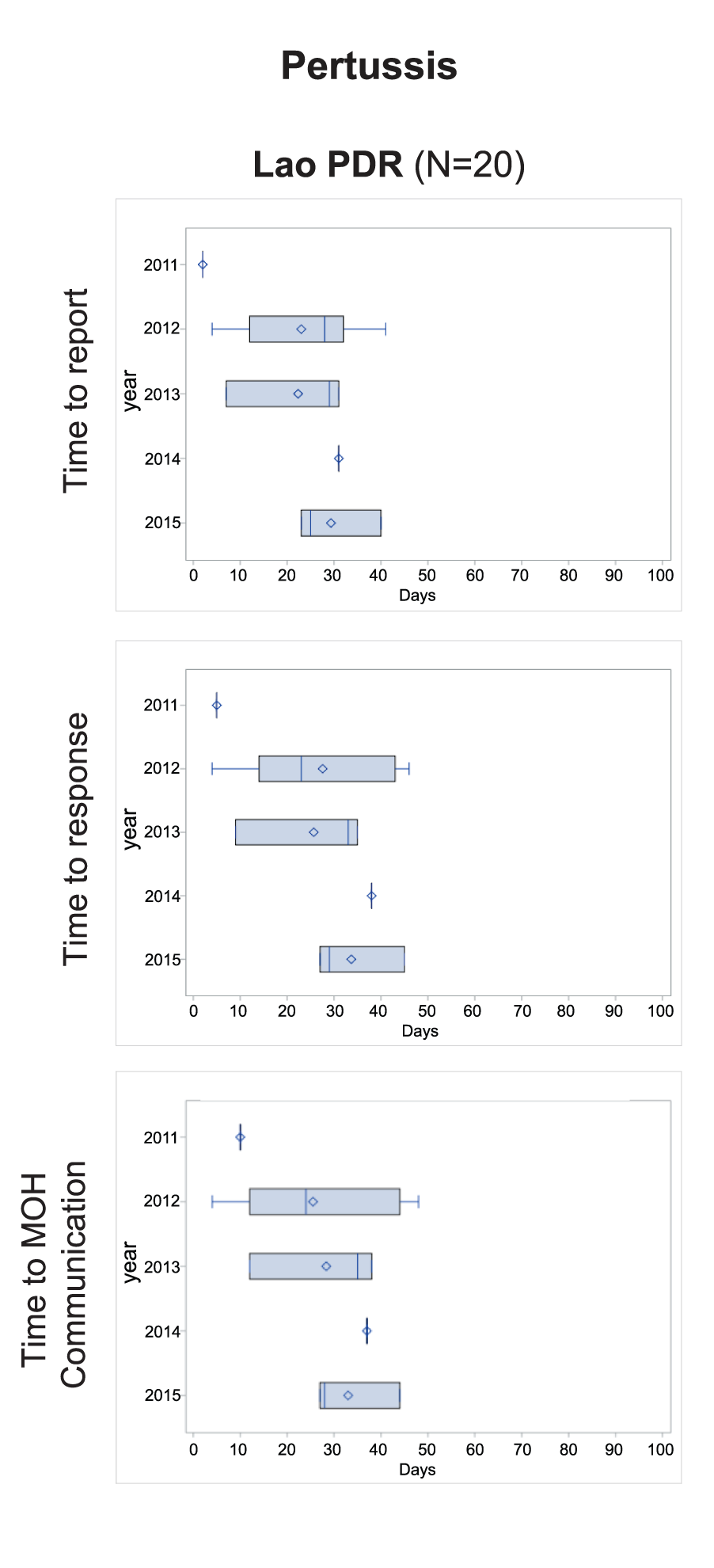

Supplement: S9 Fig — (TIF) [file pntd.0006425.s010.tif]
